# Dynamic regulation of the developmental establishment of the adult hippocampal neural stem cell pool

**DOI:** 10.4103/NRR.NRR-D-24-01581

**Published:** 2025-05-06

**Authors:** Feng Zhang, Guo-li Ming, Hongjun Song

**Affiliations:** School of Life Sciences, Nanjing University, Nanjing, Jiangsu Province, China; Department of Neuroscience and Mahoney Institute for Neurosciences, Perelman School for Medicine, University of Pennsylvania, Philadelphia, PA, USA; Department of Cell and Developmental Biology, Perelman School for Medicine, University of Pennsylvania, Philadelphia, PA, USA; Department of Psychiatry, Perelman School for Medicine, University of Pennsylvania, Philadelphia, PA, USA; Institute for Regenerative Medicine, University of Pennsylvania, Philadelphia, PA, USA; The Epigenetics Institute, Perelman School for Medicine, University of Pennsylvania, Philadelphia, PA, USA; Department of Neurosurgery, Perelman School for Medicine, University of Pennsylvania, Philadelphia, PA, USA

**Figure 1:**
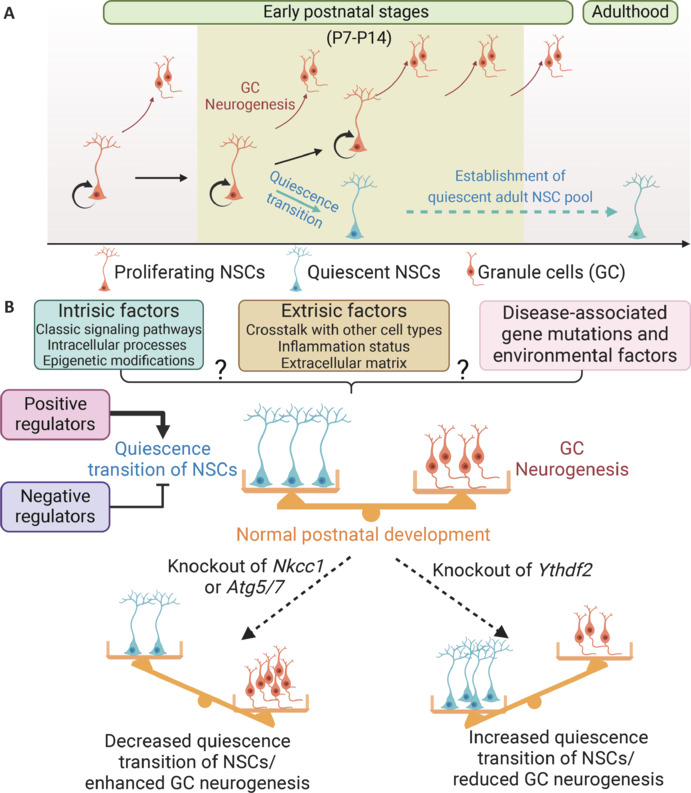
Dynamic regulation of the developmental establishment of the adult hippocampal neural stem cell pool. (A) Establishment of the quiescent adult NSC pool during postnatal development in mice. A common neural precursor population continuously contributes to the generation of the dentate GC population in the DG starting from early embryonic stages to adulthood. Furthermore, a subpopulation of proliferating DG NSCs undergoes a transition to quiescence at early postnatal stages and adopts adult NSC-like quiescent properties to establish the quiescent adult DG NSC pool. (B) The establishment of a quiescent NSC pool in the DG during the early postnatal stage is not a fixed process but instead dynamically regulated by both positive and negative regulators. Impairment in the quiescence transition of DG NSCs can result from attenuated positive factors (such as knockout of *Nkcc1* and *Atg5/7*) or negative factors (such as knockout of *Ythdf2*), which can have functional consequences on the size of the adult NSC pool and the number of developmentally generated dentate granule neurons. How this process is dynamically regulated by various intrinsic and extrinsic physiological and pathological factors needs further investigation. Created with BioRender.com. DG: Dentate gyrus; GC: granule cell; NSCs: neural stem cells.

The adult subventricular zone of the lateral ventricles and the subgranular zone in the hippocampal dentate gyrus (DG) are the two brain regions where neurogenesis occurs throughout life in the adult mammalian brain (Ming and Song, 2011). Adult quiescent hippocampal neural stem cells (NSCs) are *bona fide* stem cells and, when activated, give rise to newborn granule neurons in the adult brain, which play vital roles in learning, memory, mood, and affective cognition (Bonaguidi et al., 2011; Ming and Song, 2011). Dysregulation of this process is often associated with brain disorders in both patients and various animal models (Christian et al., 2014). The proper establishment of a quiescent adult NSC pool is essential to sustain life-long continuous neurogenesis (Urban et al., 2019). Clonal lineage-tracing in mice has identified a common neural precursor population that originates from the dentate neuroepithelium and migrates to primitive DG during the embryonic stage, and continuously contributes to the generation of dentate granule neurons from early embryonic stages to adulthood (Berg et al., 2019; **Figure 1A**). Furthermore, a subpopulation of proliferating DG NSCs undergoes a transition to quiescence during the early postnatal stage and adopts adult NSC-like quiescent properties to establish the quiescent adult DG NSC pool in mice (Berg et al., 2019; Bond et al., 2021; **Figure 1A**). Importantly, disturbances in the quiescence acquisition and fate determination of DG NSCs are closely associated with abnormal postnatal DG neurogenesis and neuronal circuitry formation during development, which would impair hippocampal function (Zhang et al., 2023, 2024; Jimenez-Cyrus et al., 2024). A better understanding of the fundamental mechanisms underlying adult neurogenesis and the establishment of a quiescent NSC pool is necessary to harness this striking intrinsic regenerative capacity in the adult mammalian brain for regeneration and repair. While the mechanisms regulating the activation and quiescence maintenance of adult DG NSCs have been widely investigated (Urban et al., 2019), research into cellular and molecular mechanisms governing the establishment of the adult NSC pool during development remains in its infancy.

Given the important function of continuous adult neurogenesis, it is generally expected that the process for the establishment of the adult NSC pool during development is precisely controlled via complex molecular programs. Several recent studies in mice have started to unravel critical molecular players for quiescence acquisition, a critical step during the establishment of the adult NSC pool at early postnatal stages (Calatayud-Baselga et al., 2023; Zhang et al., 2023; Jimenez-Cyrus et al., 2024; **Figure 1B**). For example, knockout of *Nkcc1*, a chloride transporter, suppresses the quiescence acquisition of postnatal NSCs that leads to a smaller adult NSC pool and increased generation of dentate granule neurons in the DG, possibly through decreased GABA-mediated depolarization due to reduced chloride influx and intracellular chloride concentration (Zhang et al., 2023). Similarly, knockout of *Atg5* or *Atg7*, critical components of the autophagy machinery, also leads to attenuated quiescence transition of NSCs with a reduced adult NSC pool and increased granule neuron generation in the DG (Calatayud-Baselga et al., 2023; Jimenez-Cyrus et al., 2024; **Figure 1B**). These studies highlight how various factors, including GABAergic neurotransmitter signaling and autophagy, positively regulate the quiescence acquisition of NSCs and establish a balanced pool of quiescent NSCs and neurons in the early postnatal DG.

A very recent study has changed our basic understanding of the developmental establishment of the quiescent adult NSC pool in the DG (Zhang et al., 2024). This study identified a negative regulator of the quiescence transition of DG NSCs at an early postnatal stage in mice through convergent epitranscriptomic co-regulation of multiple components within the same signaling pathway in NSCs (**Figure 1B**). Specifically, m^6^A/YTHDF2-mediated signaling promotes mRNA decay of multiple transforming growth factor-β (TGFβ) pathway components, which cell-autonomously suppress TGFβ-signaling activation in proliferating NSCs to control the balance of quiescence acquisition and neurogenesis. YTHDF2, a reader protein of m^6^A mRNA modification, which is the most abundant internal mRNA modification, promotes the decay of m^6^A-tagged mRNA. In proliferating DG NSCs, m^6^A modification of multiple components of the TGFβ signaling axis, including TGFβ ligands, receptors, maturation factors, transcription regulators, and signaling regulators, can be recognized by YTHDF2, resulting in their enhanced mRNA decay and suppression of TGFβ signaling in proliferating NSCs. Conversely, knockout of *Ythdf2*, leads to the dramatic up-regulation of TGFβ signaling activation and promotes the quiescence acquisition of early postnatal DG NSCs at the expense of reduced neurogenesis of dentate granule neurons and an enlarged quiescent NSC pool. This study reveals the first known negative regulator and gatekeeper mechanism that modulates the establishment of the quiescent NSC pool during development.

Altogether, these findings indicate that the establishment of the quiescent NSC pool in the DG during the early postnatal stage in mice is not a fixed process, but instead dynamically regulated by both positive and negative regulators, which has consequences for the size of the adult NSC pool and the number of developmentally generated dentate granule neurons. Decreased quiescence transition, which can result from dysregulated GABAergic signaling or autophagy, leads to enhanced proliferation of NSCs, increased neurogenesis, and a smaller quiescent NSC pool (Calatayud-Baselga et al., 2023; Zhang et al., 2023; Jimenez-Cyrus et al., 2024). On the contrary, increased quiescence transition, which can be induced by impaired m^6^A-mediated mRNA decay, results in reduced neurogenesis and an expanded quiescent NSC pool (Zhang et al., 2024; **Figure 1B**). These exciting advancements also raise many questions for future studies.

First, at the molecular level, beyond the reported TGFβ signaling and autophagy factors (Calatayud-Baselga et al., 2023; Jimenez-Cyrus et al., 2024; Zhang et al., 2024), it remains an open question whether other classic signaling pathways like Hippo, Notch, and mTOR signaling, various intracellular processes such as vesicle trafficking and mitochondria dynamics, as well as other epigenetic and epitranscriptomic modifications, also play critical roles in regulating the establishment of the quiescent NSC pool in the DG. Better experimental models will help this effort. For example, despite the successful application of *in vitro* cultured NSCs derived from early postnatal mouse hippocampus to study the intrinsic molecular mechanisms (Calatayud-Baselga et al., 2023; Zhang et al., 2024), there remains a significant gap between *in vitro* and *in vivo* systems. An improved *in vitro* culturing system, which can simultaneously model the proliferation, differentiation, quiescence transition, and reactivation of NSCs, will provide better opportunities for uncovering the underlying intrinsic mechanisms involved in this process.

Second, at the cellular level, beyond the intrinsic mechanisms identified so far, how interactions between proliferating NSCs and features of their local environment, such as newborn neurons, astrocytes, microglia, oligodendrocytes, vascular cells, and extracellular matrix, may modulate this process, needs detailed investigations. Cell-cell interaction analysis of published single-cell sequencing data of the developing mouse DG will help to reveal novel insights into the mechanisms underlying cell type-specific regulation of NSC quiescence transition (Hochgerner et al., 2018). As the number and gene expression pattern of different local cell types dynamically change during this period, more in-depth singl-cell genomic and spatial analyses may provide a more holistic picture of the molecular and cellular mechanisms underlying the intricate interactions during the process of establishing the adult NSC pool and a foundation for future functional analysis with specific manipulations.

Third, given the dynamic nature of the establishment of an adult NSC pool, it will be interesting to investigate how the animal’s experience and environment, such as social interactions, viral infections, as well as genetic risk factors for neurological and psychiatric disorders, may affect the balance between the establishment of the quiescent DG NSC pool and developmental DG neurogenesis. While these topics have been explored in adult hippocampal neurogenesis (Ming and Song, 2011), few studies have examined their impact on the process of establishment of the adult NSC pool. For instance, viral infection in the embryonic brain, such as ZIKA virus infection, or chronic stress of early newborn mice, such as following maternal deprivation, may lead to abnormal activation of microglia and astrocytes, resulting in altered secretion of cytokines and interactions between NSCs and activated glial cells that could affect the quiescence transition and activation of postnatal dentate NSCs. Importantly, given the close relationship between the quiescence transition of NSCs and developmental neurogenesis in the DG, dysregulation of this process could lead to abnormal granule cell generation and an altered size of the NSC pool during development, which may have lasting effects throughout life. Therefore, it will be interesting to investigate the long-term effects of changes in the balance of the adult NSC pool and the developmental generation of dentate granule neurons, including the impact on learning, memory, and mood regulation as well as potential contributions to the pathogenesis of brain disorders.

Fourth, it remains to be determined whether the dynamic regulation of the adult NSC pool is a conserved mechanism across brain regions and species. For example, NSCs from adult subventricular zone are also established via quiescence transition during embryonic stages in mice, whereas little is known about the establishment of the adult NSC pool during human hippocampal development (Bond et al., 2021). The significant differences between mouse and human brain development—such as neurogenesis rates and tempo, brain size and complexity, and developmental timelines—make it challenging to directly translate findings from mouse studies to human contexts. Indeed, a recent analysis of molecular characteristics of immature neurons in the adult dentate gyrus across mice, pigs, macaques, and humans found divergent gene expression patterns but convergent biological processes, highlighting the importance of conducting independent molecular and functional analyses for adult neurogenesis in different species (Zhou et al., 2025). Future single-cell RNA sequencing and spatial transcriptomic analysis of fetal human dentate development may reveal the molecular cascade and cellular mechanisms underlying the transition of proliferating NSCs into quiescent NSCs in the developing human DG and the establishment of the adult NSC niche. In turn, these results will guide the development of human pluripotent stem cell-based hippocampal organoid models for molecular and functional studies.

In summary, the molecular and cellular mechanisms controlling the establishment of the quiescent adult NSC pool have just begun to be unraveled. Advances in this field will be valuable for developing pharmacological approaches to expand the adult quiescent NSC pool, thereby enhancing adult neurogenesis while preserving normal developmental neurogenesis, which holds significant therapeutic potential for regeneration and repair in the adult human brain.

*The research in Drs. Ming and Song laboratories was supported by National Institutes of Health (R35NS137480, R35NS116843, and RF1AG079557), and by Dr. Miriam and Sheldon G. Adelson Medical Research Foundation.*
